# Population-based case–control study of soyfood intake and breast cancer risk in Shanghai

**DOI:** 10.1054/bjoc.2001.1873

**Published:** 2001-08

**Authors:** Q Dai, X-O Shu, F Jin, J D Potter, L H Kushi, J Teas, Y-T Gao, W Zheng

**Affiliations:** Vanderbilt-Ingram Cancer Center and Department of Medicine, Vanderbilt University, Nashville, TN, 37232–8300; Department of Epidemiology, Shanghai Cancer Institute, Shanghai, 200032, P.R. China; Cancer Prevention Research Program, Fred Hutchinson Cancer Research Center, Seattle, WA, 98109–1024; Columbia University, New York, NY, 10027; School of Public Health, University of South Carolina, SC, 29203

**Keywords:** breast cancer, body mass index, ER and PR, soyfood

## Abstract

We evaluated the association of soyfood intake and breast cancer risk in a population-based case–control study among Chinese women in Shanghai. Included in the study were 1459 cases and 1556 age-matched controls, with respective response rates of 91.1% and 90.3%. Usual soyfood intake was assessed using a food frequency questionnaire (FFQ). Separate analyses were performed for all subjects and for the subset who reported no recent change in soyfood intake. The intake levels of soyfoods among women in Shanghai are high, with 96.6% women reporting soyfood consumption at least once a week. A statistically non-significant reduced risk (odds ratio (OR) = 0.78 95% CI = 0.52–1.16) of breast cancer was observed among those who reported eating soyfood at least once a week. Compared to those in the lowest decile intake group, women in the highest decile intake group had a 30% reduced risk of breast cancer (OR = 0.66, 95% CI = 0.46–0.95), but no monotonic dose–response relation was observed (*P* for trend, 0.28). Stratified analyses showed that the inverse association was restricted primarily among women who had a high body mass index (BMI), with an adjusted OR of 0.30 (95% CI = 0.10–0.94) observed for the highest intake group. The reduction in risk was stronger for breast cancer positive for both oestrogen receptor (ER) and progesterone receptor (PR) (OR = 0.44, 95% CI = 0.25–0.78) than those with other ER/PR status. More pronounced inverse associations were observed in analyses among those who reported no recent change in soyfood intake than those conducted in all subjects. A dose–response relation between soyfood intake and breast cancer risk was observed in this subset of women (*P* for trend, 0.02), with an OR of 0.46 (95%CI = 0.28–0.75) for those in the highest decile intake group. No clear monotonic dose–response relation was found between soyfood intake and breast cancer risk among regular soy eaters, but nevertheless the results suggest that regular soyfood consumption may reduce the risk of breast cancer, particularly for those positive for ER and PR; the effect may be modified by body mass index. © 2001 Cancer Research Campaign http://www.bjcancer.com


*British Journal of Cancer* (2001) **85**(3), 372–378
© 2001 Cancer Research Campaign
doi: 10.1054/ bjoc.2001.1873, available online at http://www.idealibrary.com on IDEAL®

http://www.bjcancer.com

### Population-based case–control study of soyfood intake and breast cancer risk in Shanghai

**Q Dai[1,5], X-O Shu[1], F Jin[2], JD Potter[3], LH Kushi[4], J Teas[5], Y-T Gao[2] and W Zheng[1]**

[1]Vanderbilt-Ingram Cancer Center and Department of Medicine, Vanderbilt University, Nashville, TN 37232–8300; [2]Department of Epidemiology, Shanghai Cancer Institute, Shanghai 200032, P.R. China; [3]Cancer Prevention Research Program, Fred Hutchinson Cancer Research Center, Seattle, WA 98109–1024; [4]Columbia University, New York, NY 10027; [5]School of Public Health, University of South Carolina, SC 29203

**Summary** We evaluated the association of soyfood intake and breast cancer risk in a population-based case–control study among Chinese women in Shanghai. Included in the study were 1459 cases and 1556 age-matched controls, with respective response rates of 91.1% and 90.3%. Usual soyfood intake was assessed using a food frequency questionnaire (FFQ). Separate analyses were performed for all subjects and for the subset who reported no recent change in soyfood intake. The intake levels of soyfoods among women in Shanghai are high, with 96.6% women reporting soyfood consumption at least once a week. A statistically non-significant reduced risk (odds ratio (OR) = 0.78 95% CI = 0.52–1.16) of breast cancer was observed among those who reported eating soyfood at least once a week. Compared to those in the lowest decile intake group, women in the highest decile intake group had a 30% reduced risk of breast cancer (OR = 0.66, 95% CI = 0.46–0.95), but no monotonic dose–response relation was observed (*P* for trend, 0.28). Stratified analyses showed that the inverse association was restricted primarily among women who had a high body mass index (BMI), with an adjusted OR of 0.30 (95% CI = 0.10–0.94) observed for the highest intake group. The reduction in risk was stronger for breast cancer positive for both oestrogen receptor (ER) and progesterone receptor (PR) (OR = 0.44, 95% CI = 0.25–0.78) than those with other ER/PR status. More pronounced inverse associations were observed in analyses among those who reported no recent change in soyfood intake than those conducted in all subjects. A dose–response relation between soyfood intake and breast cancer risk was observed in this subset of women (*P* for trend, 0.02), with an OR of 0.46 (95%CI = 0.28–0.75) for those in the highest decile intake group. No clear monotonic dose–response relation was found between soyfood intake and breast cancer risk among regular soy eaters, but nevertheless the results suggest that regular soyfood consumption may reduce the risk of breast cancer, particularly for those positive for ER and PR; the effect may be modified by body mass index. © 2001 Cancer Research Campaign http://www.bjcancer.com

**Keywords**: breast cancer; body mass index; ER and PR; soyfood

Historically, the incidence rates of breast cancer in China, Japan and some other Asian countries have been substantially lower than those in the United States and Europe (Parkin et al, 1997). Ecological and migration studies have suggested that the marked international variation in breast cancer incidence largely due to environmental factors, such as dietary habits (Henderson et al, 1996). Soyfoods, consumed in high quantities by Asian women, are one possible explanation for this difference. Soyfoods and some of their constituents have been implicated in numerous in vitro and animal studies as being potentially protective against breast and perhaps other hormone-related cancers (Adlercreutz and Mazur, 1997; Messina et al, 1997). Analytic epidemiologic studies on soyfood intake and breast cancer risk, however, have been few and inconsistent (Hirohata et al, 1985; Hirayama 1990; Lee et al, 1991, 1992; Hirose et al, 1995; Yuan et al, 1995; Greenstein et al, 1996; Wu et al, 1996, 1998; Witte et al, 1997; Key et al, 1999). Part of the inconsistency may be due to errors in exposure assessment and confounding. Intakes of soyfood in the United States are low, limiting the ability to conduct informative observational studies (Wu et al, 1998).

*Received 2 January 2001*
*Revised 9 April 2001*
*Accepted 10 April 2001*

*Correspondence to:* W Zheng

Soyfoods are the major sources of isoflavones, a group of phytoestrogens showing both weak oestrogenic and antioestrogenic activities. These phytochemicals have been shown to compete with endogenous oestrogens for oestrogen receptors in many in vitro and in vivo systems (Adlercreutz and Mazur, 1997). High soy consumption has been shown in some studies to decrease ovarian hormones (Lu et al, 2000) and increase plasma sex hormone-binding globulin (SHBG) (Duncan et al, 1999; Pino et al, 2000). Thus the potential cancer-inhibitory effect of soyfood may be more evident for breast cancers expressing oestrogen receptor. In addition to an anti-oestrogenic effect, several recent animal studies showed that soy proteins improve glucose tolerance and insulin sensitivity (Wagner et al, 1997). Insulin resistance has been linked to an elevated risk of breast cancer (Bruning et al, 1992; Yang et al, 2001). Body weight, measured using body mass index (BMI), and central obesity measured using the waist-to-hip ratio (WHR) are associated with an elevated level of blood insulin and a reduced level of SHBG (and thus an elevated level of oestrogen bioavailability) (Pasquali et al, 1987; Seidell, 2000). BMI is also positively associated with oestrogen levels among postmenopausal women (Siiteri, 1987). A protective effect of soyfood intake may therefore be more pronounced among women with a high BMI and/or WHR. To evaluate the association of soyfood intake with breast cancer risk and potential modifying effects of BMI, WHR and ER/PR status, we analysed data from the Shanghai Breast

Cancer Study, a population-based case–control study of breast cancer among Chinese women in Shanghai during 1996–1998.

### METHODS

The Shanghai Breast Cancer Study was designed to recruit all women aged 25 to 64 newly diagnosed with breast cancer from August 1996 to March 1998. All study subjects were permanent residents of urban Shanghai with no prior history of cancer. The ethical aspects of the study were approved by relevant institutional review boards. Through a rapid case-ascertainment system, supplemented by the population-based Shanghai Cancer Registry, 1602 eligible breast cancer cases were identified during the study period, and in-person interviews were completed for 1459 (91.1%). The major reasons for non-participation were refusal (109 cases, 6.8%), death prior to interview (17 cases, 1.1%), and inability to locate (17 cases, 1.1%). 2 senior pathologists reviewed all slides to confirm all cancer diagnoses. Detailed information on cancer diagnosis and treatment including oestrogen (ER) and progesterone (PR) status was abstracted from medical charts. The information on ER/PR status was obtained for 956 of the 1459 breast cancer cases. Of these, 52.7% were ER+/PR+; 11.2%, ER+/PR−; 10.6%, ER−/PR+; and 25.5%, ER−/PR−.

The Shanghai Resident Registry, which keeps records for all permanent residents in urban Shanghai, was used to select controls randomly from female residents, frequency-matched to cases by age (5-year interval). The number of controls in each age-specific stratum was determined in advance according to the age distribution of the incident breast cancer cases reported to the Shanghai Cancer Registry from 1990–1993. Only women who lived at the address identified during the study period were considered to be eligible for the study. In-person interviews were completed for 1556 (90.3%) of the 1724 eligible controls identified. Reasons for non-participation included refusal (166 controls, 9.6%), and death prior to the interview (2 controls, 0.1%).

All study participants were interviewed in person by trained interviewers and measured for weight, circumferences of waist and hip, and sitting and standing heights. A structured questionnaire was used to elicit detailed information on demographic factors, menstrual and reproductive history, hormone use, dietary habits, prior disease history, physical activity, tobacco and alcohol use, weight and family history of cancer. Information on usual dietary intake was collected using a comprehensive quantitative food-frequency questionnaire (FFQ), including questions usual consumption of soymilk, tofu, dry soybeans, soy products other than tofu, fresh soybeans and soybean sprouts. These food items were shown to account for over 90% of soyfoods consumed in Shanghai in a recent 24-hour dietary survey (Satia et al, 1999).

During the interview, each study participant was first asked how frequently she consumed a specific soyfood or group of soyfoods (per day, week, month, year or never), followed by a question on how many lians (= 50 grams) she ate the food(s) per unit of time (day, week, month, or year) in the previous 5-year period, ignoring any recent changes. For fresh soybeans, subjects were asked to describe their consumption when the food was available on the market. 7 months after initiation of the study, a supplementary survey was added, in which all subjects were asked whether their intake levels of selected foods (including soyfoods) 1 week before interview were similar, increased or decreased compared to their usual intake levels of these foods in the past 5 years. The supplementary survey was completed for 1104 cases and 1232 controls. 65.8% of cases and 82.5% of controls reported no change in soyfood intake; 16.9% of cases versus 7.2% of controls reported an increase and 16.8% of cases versus 10.0% of controls reported a decrease in soyfood intake.

Total soyfood consumption was measured by summing the soy protein derived from soyfoods. Soy protein intake was computed based on the Chinese Food Composition Table (Chinese Academy of Medical Sciences, 1991). Total isoflavone intake was also calculated using the published data (Chen et al, 1999). Soy protein and soy isoflavones are better measurements of total soyfood intake because individuals who rarely eat one type of soyfood might eat other types frequently and soy protein concentrations vary widely across different soyfoods. Because results from our analyses showed similar patterns using soy protein and soy isoflavone intakes and some recent studies suggested that soy, but not isoflavones, may be more important in reducing breast cancer risk (Constantinou et al, 2000), we present results only on soy protein. Partial sum-of-squares in the multivariate linear regression model was used to estimate how each individual soyfood accounted for the percentage of between-subject variation of total soy protein. Subjects who ate soyfoods less often than once per week were used as the reference in our primary analyses and we classified the regular soy consumers into 4 groups based on the quartile distributions among controls. Analyses were also conducted based on decile distributions of soyfood intake among controls.

Unconditional logistic regression models were used to obtain maximum likelihood estimates of the odds ratios (OR) and their 95% confidence intervals (95% CI), after adjusting for potential confounding variables (Breslow and Day, 1980). Age was included as a continuous variable throughout, and categorical variables were treated as dummy variables in the model. Testing for linear trend was performed by entering soyfood protein intake as continuous variable in the logistic models. Because the recall of usual diet is heavily influenced by current dietary intake (Hankinson et al, 1998), we also performed analyses among those who reported no change in soyfood intake one week before interview, in order to minimize potential misclassification errors. Further analyses were stratified by menopausal status, BMI, WHR and ER/PR status. The 75th percentile of BMI and WHR among controls was used as a cut-point in these stratified analyses, as Chinese women are, in general, thinner than Caucasian women. All statistical tests were based on 2-sided probability.

### RESULTS

Table 1 shows comparisons of cases and controls on demographic and lifestyle risk factors of breast cancer, including dietary factors. Data are presented for all subjects and for those who reported no recent change in soyfood intake, between which there were no appreciable differences. For both groups, compared to controls, cases were slightly older, had earlier menarcheal age, later menopausal age, later age at first live birth, and ate more meats and fish. Compared to controls, cases were more likely to have a higher education, a family history of breast cancer among first-degree relatives, a history of breast fibroadenoma, a higher BMI, or WHR, and were less likely to exercise regularly. All the above variables were considered potential confounders and adjusted for in subsequent analyses. No significant differences between cases and controls were observed for parity, months of breast feeding, alcohol consumption, use of oral contraceptives, hormone replacement therapy, measured height, and usual intake of total energy, fruits and vegetables and fat.

*© 2001 Cancer Research Campaign*

**Table 1** Comparison of cases and controls on selected demographics and breast cancer risk factors, the Shanghai Breast Cancer Study, 1996–1998

| | All subjects | | | Subjects with no dietary changee | | |
|---|---|---|---|---|---|---|
| | Cases[a] (*n* = 1459) | Controls[a] (*n* = 1556) | *P* value | Cases[a] (*n* = 724) | Controls[a] (*n* = 1015) | *P* value |
| Age | 47.8±8.0 | 47.2±8.8 | 0.03 | 47.8±8.04 | 7.0±8.5 | 0.06 |
| Education (%) | | | | | | |
| No formal education | 3.6 | 5.5 | | 3.0 | 4.7 | |
| Elementary school | 8.5 | 8.4 | | 8.7 | 8.2 | |
| Middle and high school | 74.3 | 75.4 | | 74.5 | 77.1 | |
| College and higher | 13.6 | 10.7 | 0.01 | 13.8 | 10.0 | 0.04 |
| Breast cancer in first degree relatives (%) | 3.7 | 2.4 | 0.05 | 3.4 | 2.2 | 0.10 |
| Ever had breast fibroadenoma (%) | 9.6 | 5.0 | <0.01 | 10.2 | 5.4 | <0.01 |
| Regular alcohol drinke r(%) | 4.0 | 4.1 | 0.99 | 4.1 | 4.0 | 0.92 |
| Ever used oral contraceptives (%) | 21.9 | 20.9 | 0.51 | 20.9 | 20.4 | 0.77 |
| Ever used hormone replacement therapy (%) | 2.9 | 2.7 | 0.76 | 2.2 | 2.9 | 0.40 |
| Exercised regularly (%) | 18.8 | 25.2 | <0.01 | 17.4 | 22.8 | <0.01 |
| Height (cm) | 158.9±5.10 | 158.5±5.37 | 0.07 | 158.8±4.90 | 158.6±5.46 | 0.39 |
| Body mass index | 23.5±3.4 | 23.1±3.4 | <0.01 | 23.6±3.5 | 23.2±3.3 | 0.02 |
| Waist-to-hip ratio | 0.81±0.06 | 0.80±0.06 | <0.01 | 0.81±0.05 | 0.80±0.06 | <0.01 |
| Nulliparous (%) | 5.1 | 3.9 | 0.13 | 4.8 | 3.8 | 0.32 |
| Number of live births[b] | 1.5±0.85 | 1.5±0.86 | 0.54 | 1.5±0.81 | 1.5±0.82 | 0.55 |
| Age at first live birth[b] (years) | 26.8±4.2 | 26.2±3.9 | <0.01 | 25.6±7.1 | 25.3±6.2 | 0.30 |
| Months of breast feeding[c] | 15.1±13.1 | 15.9±14.0 | 0.81 | 11.3±12.3 | 12.0±12.8 | 0.23 |
| Menarcheal age (years) | 14.5±1.6 | 14.7±1.7 | <0.01 | 14.4±1.6 | 14.7±1.7 | <0.01 |
| Menopausal age[d](years) | 48.1±4.6 | 47.5±4.9 | 0.02 | 47.8±4.6 | 47.2±5.1 | 0.21 |
| Energy intake (kcal/day) | 1865.9±464.2 | 1839.9±464.2 | 0.12 | 1816.3±446.3 | 1803.2±445.5 | 0.95 |
| Total fat intake (g/day) | 36.3±17.4 | 35.3±16.2 | 0.08 | 34.5±16.1 | 34.6±15.5 | 0.90 |
| Total meat intake (g/day) | 49.6±34.5 | 44.1±28.4 | <0.01 | 47.3±30.8 | 45.8±27.2 | 0.03 |
| Total fruit and vegetable (g/day) | 290.4±160.7 | 288.7±165.0 | <0.78 | 302.1±166.5 | 289.9±157.6 | 0.21 |
| Total fish intake (g/day) | 35.3±36.7 | 30.2±30.0 | <0.01 | 33.4±30.6 | 29.1±26.6 | <0.01 |

Subjects with missing values were excluded from the analysis. [a]Unless otherwise specified, mean±SD are presented. [b]Among women who had live births. [c]Among women who ever breast fed. [d]Among menopausal women. [e]Out of 1104 cases and 1232 controls who completed the supplementary questionnaire which was added to the study 7 months after the initiation of the study–see text.

**Table 2** Intake levels of soybean food and soy protein among controls in the Shanghai Breast Cancer Study, 1996–1998

| | Mean±SD | Median (25th, 75th percentile) | Protein equivalent Mean±SD |
|---|---|---|---|
| Soy foods (g/week) | | | |
| Total soyfood | 947.8±889.0 | 654.5 (350.0, 1249.5) | |
| Soy milk | 405.3±683.2 | 0 (0, 466.9) | 7.0±12.6 |
| Tofu | 223.3±241.5 | 186.9 (93.1, 373.1) | 14.0±14.7 |
| Processed soy products other than tofu | 156.8±534.8 | 93.1 (25.9, 233.1) | 38.5±45.5 |
| Dry soybean seed | 7.0±51.1 | 0 (0, 2.8) | 2.1±17.5 |
| Fresh soybean | 117.6±152.6 | 70.0 (30.8, 155.4) | 8.4±10.5 |
| Soybean sprout | 37.8±60.9 | 11.9 (0, 46.9) | 1.4±2.8 |
| Soy protein (g/week) | 72.1±70.7 | 56.0 (33.6, 90.3) | |
| Isoflavones (mg/week) | 286.3±276.5 | 232.4 (130.9, 373.1) | |

Summarized in Table 2 are the intakes of soyfoods, soy protein and isoflavones among the control group. Mean intakes for soy protein and isoflavone were 72.1 g week$^{-1}$ and 286.3 mg week$^{-1}$, respectively. Processed soy products other than tofu accounted for more than half of total soy protein intake. Tofu, the second most commonly consumed soy product in the population accounted for 19.4% of soy protein intake. Soy protein was positively correlated with intake of meats ($r$ = 0.11, $P$ < 0.001), fish ($r$ = 0.30, $P$ < 0.001), and fruits and vegetables ($r$ = 0.27, $P$ < 0.001), although the correlation was only weak to moderate (data not shown in tables). These dietary variables were also adjusted in data analyses.

The intake levels of soyfoods were high in Shanghai women, with 96.2% cases and 96.8% controls reporting soyfood consumption at least once a week. After adjusting for total energy and other potential confounders, the OR for breast cancer was 0.78 (95% CI: 0.52–1.16) for regular soyfood consumers (at least once a week) compared to those who ate soyfoods less often than weekly (Table 3). Among regular soyfood eaters, a 30% (95% CI = 0.43–1.02) reduced risk appeared among women with the highest soy protein intake but the test for trend was not significant ($P$ = 0.10). A similar pattern of associations was observed among women who reported no recent change in soyfood intake, although the inverse association was more pronounced ($P$ for trend, 0.02). Analyses according to the decile distribution of soy protein intake among controls showed a similar pattern of inverse associations, and the ORs were statistically significant among women with the highest intake of soy protein. The inverse association was observed in both pre-and postmenopausal women (data not shown).

© 2001 Cancer Research Campaign

**Table 3** Adjusted odds ratios (ORs) and 95% confidence intervals (CIs) for the association of breast cancer with soy protein, the Shanghai Breast Cancer Study, 1996–1998

| Soy protein (g/week) | All subjects | | Subjects without dietary change | |
|---|---|---|---|---|
| | Cases/controls | OR* (95%CI) | Cases/controls | OR[a] (95%CI) |
| | Analyses using occasional soyfood eaters as the reference group | | | |
| Occasionally[b] | 56/48 | 1.00 | 29/33 | 1.00 |
| Weekly | 1403/1508 | 0.78 (0.52–1.16) | 697/983 | 0.79 (0.47–1.33) |
| ≤35.0 | 322/378 | 0.76 (0.49–1.16) | 174/255 | 0.81 (0.47–1.42) |
| ≤58.8 | 366/376 | 0.82 (0.54–1.26) | 203/242 | 0.93 (0.54–1.63) |
| ≤91.0 | 388/374 | 0.88 (0.58–1.36) | 188/247 | 0.89 (0.51–1.55) |
| >91.0 | 327/380 | 0.66 (0.43–1.02) | 132/239 | 0.56 (0.32–1.00) |
| Trend test | | $P$ = 0.10 | | $P$ = 0.02 |
| | Analyses using by decile distribution of soy protein intake among controls | | | |
| ≤18.6 | 156/156 | 1.00 | 86/102 | 1.00 |
| ≤28.7 | 122/157 | 0.74 (0.53–1.03) | 67/116 | 0.61 (0.39–0.93) |
| ≤37.3 | 121/154 | 0.71 (0.51–1.00) | 59/97 | 0.63 (0.40–0.99) |
| ≤47.0 | 170/157 | 0.98 (0.71–1.36) | 104/103 | 1.05 (0.70–1.59) |
| ≤56.4 | 135/154 | 0.80 (0.58–1.12) | 73/98 | 0.74 (0.48–1.15) |
| ≤68.9 | 188/157 | 1.14 (0.83–1.57) | 90/105 | 0.93 (0.61–1.42) |
| ≤82.8 | 159/157 | 0.91 (0.66–1.27) | 72/97 | 0.77 (0.50–1.20) |
| ≤102.7 | 157/153 | 0.89 (0.64–1.24) | 78/108 | 0.73 (0.47–1.12) |
| ≤139.1 | 120/156 | 0.65 (0.46–0.92) | 47/95 | 0.47 (0.29–0.76) |
| >139.1 | 131/155 | 0.66 (0.46–0.95) | 50/95 | 0.46 (0.28–0.75) |
| Trend test | | 0.28 | | 0.02 |

[a]Adjusted for age, education, first degree family history of breast cancer, history of breast fibroadenoma, waist-to-hip ratio, age at menarche, physical activity, birth of ≥ 1 child, age at first birth, menopausal status, age at menopause, intake of meats, fish, and total energy. [b]Refers to those who ate soyfood less than once per week.

Analyses were performed by ER/PR status of breast cancer and stratified by BMI or WHR levels (Table 4). A 60% reduced risk was observed for ER+/PR+ breast cancer among regular soy consumers, and the test for trend was statistically significant ($P$ for trend, 0.05). This association was more marked among those who reported no recent change in soyfood intake ($P$ for trend, 0.004), with an OR of 0.28 (95%CI = 0.13–0.57) observed in the highest intake group. In contrast, no apparent association of soyfood intake was observed for other groups of breast cancer cases defined by ER/PR status. In analyses stratified by BMI level, a substantially reduced risk (60–70%) of breast cancer was observed for those who had a BMI of 25 or higher. This inverse association was more evident among those who had no recent change in soyfood intake ($P$ for trend, 0.03) with an OR of 0.21 (95% CI 0.06–0.77) for the highest intake group. The risk was only slightly reduced among regular soy consumers who had a low BMI. This pattern of association suggests an interaction, although the tests for multiplicative interaction were not statistically significant ($P$ = 0.51 for all subjects; $P$ = 0.14 for those who had no recent change). The inverse association with soyfood intake also appears to be stronger among women with a high WHR than those with a low WHR, especially among women who reported no recent change in soyfood intake.

### DISCUSSION

The results of this large population-based case–control study suggest that regular soyfood intake, particularly very high intake of soyfoods, may be associated with a reduced risk of breast cancer, particularly those positive for both ER and PR. The inverse association was more evident among women who had a high BMI than those who had a low BMI. These findings are consistent with observations from in vitro and animal studies implicating potential cancer inhibitory effects of soy and its constituents (Adlercreutz and Mazur, 1997).

The primary concern in this study is the possibility that potential errors, both differential and non-differential, in assessing soyfood intake may have biased results. The diets of cases may have changed as a result of the cancer diagnosis and treatment (Willett, 1998). Current diet may also strongly influence the recall of usual diet (Willett, 1998). Previous studies showed that breast cancer cases who were receiving chemotherapy during the interview reported a higher intake of micronutrients, macronutrients and calories than cases who did not receive chemotherapy (Potischman et al 1997, 1999). In our study, through a rapid case-reporting system, we were able to interview nearly half of cases before they received any treatment, and recent dietary changes for these women were less substantial than those who had completed cancer treatments. We found that there was no apparent association of usual soyfood intake with breast cancer risk among women who reported a recent increase in their soyfood intake (34% of cases and 18% of controls). It is likely that some cases may have increased their soyfood intake after breast cancer diagnosis and treatment, and the recent dietary change may have affected the recall of usual diet in some patients. Such a recall bias may have biased the true inverse association towards the null in the analysis of all subjects combined. Non-differential errors are a concern in epidemiologic studies. This error may be particularly significant among those who reported a recent dietary change, an indication of unstable dietary practices. It has been shown that non-differential misclassification usually attenuates the true association. In order to minimize the potential influence of measurement errors, both differential and non-differential, we asked study subjects to report their recent dietary practice and performed analyses on the

© 2001 Cancer Research Campaign

**Table 4** Adjusted odds ratios (ORs) and 95% confidence intervals (Cls) for the association of breast cancer with soy protein by ER/PR, BMI, WHR status, the Shanghai Breast Cancer Study, 1996–1998

| Stratifying variables | No of cases/ controls | | All subjects – Soy protein intake by quartile (g/week) | | | | *P* for trend | No of cases/ controls | Subjects with no soyfood intake change – Soy protein intake by quartile (g/week) | | | | | *P* for trend |
|---|---|---|---|---|---|---|---|---|---|---|---|---|---|---|
| | | <Weekly | Q1 | Q2 | Q3 | Q4 | | | <Weekly | Q1 | Q2 | Q3 | Q4 | |
| **ER/PR status** | | | | | | | | | | | | | | |
| ER+/PR+ | 504/1556 | 1.00 | 0.51 (0.29–0.87) | 0.52 (0.30–0.89) | 0.65 (0.38–1.12) | 0.44 (0.25–0.78) | 0.05 | 244/1016 | 1.00 | 0.44 (0.23–0.88) | 0.49 (0.25–0.97) | 0.56 (0.29–1.10) | 0.28 (0.13–0.57) | 0.004 |
| ER-/PR- | 244/1556 | 1.00 | 0.94 (0.42–2.14) | 1.30 (0.58–2.91) | 1.09 (0.48–2.47) | 0.78 (0.33–1.81) | 0.91 | 130/1016 | 1.00 | 1.04 (0.34–3.23) | 1.44 (0.47–4.40) | 1.15 (0.37–3.58) | 0.69 (0.21–2.25) | 0.62 |
| ER+/PR- or ER-/PR+ | 208/1556 | 1.00 | 1.60 (0.55–4.67) | 1.68 (0.58–4.90) | 1.72 (0.59–5.02) | 1.58 (0.53–4.66) | 0.41 | 102/1016 | 1.00 | 1.22 (0.34–4.37) | 1.17 (0.32–2.23) | 0.97 (0.26–3.58) | 0.75 (0.20–2.88) | 0.43 |
| **BMI (by 75th percentile**) | | | | | | | | | | | | | | |
| BMI<25 | 1016/1154 | 1.00 | 0.86 (0.53–1.38) | 0.97 (0.60–1.56) | 0.96 (0.60–1.55) | 0.76 (0.46–1.25) | 0.49 | 495/751 | 1.00 | 1.04 (0.55–1.98) | 1.17 (0.62–2.23) | 1.05 (0.55–2.00) | 0.81 (0.41–1.59) | 0.28 |
| BMI≥25 | 443/402 | 1.00 | 0.34 (0.11–1.06) | 0.34 (0.11–1.04) | 0.45 (0.15–1.39) | 0.30 (0.10–0.94) | 0.15 | 231/265 | 1.00 | 0.35 (0.10–1.25) | 0.43 (0.12–1.54) | 0.45 (0.13–1.59) | 0.21 (0.06–0.77) | 0.03 |
| **WHR (75th percentile)** | | | | | | | | | | | | | | |
| WHR<0.84 | 1021/1167 | 1.00 | 0.80 (0.49–1.30) | 0.81 (0.50–1.33) | 0.86 (0.53–1.41) | 0.72 (0.43–1.19) | 0.29 | 521/766 | 1.00 | 0.99 (0.53–1.83) | 0.99 (0.53–1.85) | 1.01 (0.54–1.89) | 0.73 ) (0.38–1.41) | 0.12 |
| WHR≥0.84 | 438/389 | 1.00 | 0.75 (0.31–1.86) | 0.96 (0.39–2.35) | 1.07 (0.44–2.61) | 0.61 (0.25–1.51) | 0.20 | 205/250 | 1.00 | 0.55 (0.15–2.11) | 1.02 (0.27–3.82) | 0.83 (0.22–3.12) | 0.35 (0.09–1.34) | 0.12 |

Adjusted for age, education, first degree family history of breast cancer, history of breast fibroadenoma, waist-to-hip ratio, age at menarche, physical activity, birth of ≥ 1 child, age at first live birth, menopausal status, age at menopause, intake of total meats, total fish, and total energy.

© 2001 Cancer Research Campaign subset of participants who reported no recent dietary change. As expected, the inverse association with soyfood intake was more evident in this group of women than all subjects combined. This finding points to the importance of controlling measurement errors in epidemiologic studies and suggests that null associations observed in epidemiologic studies, particularly case–control studies in which cases are interviewed several months after cancer diagnosis, should be interpreted with caution.

The inverse association between soyfood intake and breast cancer risk is supported by findings from ecological studies and a large body of cell culture and animal experiments (Adlercreutz and Mazur, 1997). Results from previous analytical epidemiologic studies, however, are few and inconsistent (Lee et al, 1991, 1992; Hirose et al, 1995; Yuan et al, 1995; Wu et al, 1996, 1998; Witte et al, 1997; Hirayama, 1990; Greenstein et al, 1996; Key et al, 1999). A weak but statistically non-significant inverse association was reported in 3 cohort studies (Hirayama, 1990; Greenstein et al, 1996; Key et al, 1999) and 2 case–control studies (Hirose et al, 1995; Witte et al, 1997). Only 2 studies have reported a statistically significant inverse association with soyfood intake, but neither evaluated potential modifying effects of BMI, WHR, and ER/PR status (Lee et al, 1991; Wu et al, 1996). The findings of our study are inconsistent with an earlier study conducted in Shanghai (Yuan et al, 1995) in which the FFQ included only soymilk, tofu and vegetarian chicken (a soy product) with a total estimated soy protein intake of 3.5 g $day^{-1}$ (Yuan et al, 1995). This level is only about one-third of the total soy protein intake reported in the current study (Table 2) in which tofu accounted for only 4.3% of the between-person variation in total soy protein intake in Shanghai, implying that soyfood intake in the previous Shanghai study was underestimated.

Soyfood intake is substantially lower in the US population (Greenstein et al, 1996; Wu et al, 1998), where choices of soyfoods are limited and tofu is usually the major provider of soy protein. No previous studies were specifically designed to investigate the association of soyfood intake with breast-cancer risk, and none of the dietary instruments was formally validated for measuring soyfood intake. Only one study used soy protein as a measurement of total soyfood intake (Yuan et al, 1995). Some of these limitations may account for the inconsistent findings reported from previous studies.

The Shanghai Breast Cancer Study was designed specifically to test the hypothesis that soyfood intake reduces the risk of breast cancer. The questionnaire included over 90% of the common soyfoods consumed by Shanghai residents based on the results from a 24-hour dietary recall study in a random sample of Shanghai residents (Satia et al, 1999). In a recently completed validation study of about 200 Shanghai women with 24 days (twice a month) of 24-hour dietary recalls, we found that the intake level of soy protein derived from the FFQ was correlated well with that derived from the 24-hour dietary survey ($r = 0.49$, $P < 0.001$). Consumption of soyfoods is a personal dietary preference, and intakes for most individuals are likely to be relatively stable over time, particularly in Shanghai, an area with an abundant supply of soyfoods. Using a subset of the control group in the Shanghai Breast Cancer Study, we found a clear dose–response relationship between usual soyfood protein intake derived from the FFQ and the excretion rate of isoflavonoids in an overnight urine sample (Chen et al, 1999), providing additional assurance that our dietary questionnaire is valid in capturing usual soyfood intake in the Shanghai population. We also found that breast cancer cases had a substantially low-excretion of urinary isoflavonoids than controls in a small case–control study (Zheng et al, 1999).

One puzzling finding of this study is a lack of an apparent monotonic dose–response association between soyfood intake and breast cancer among regular soy consumers in most analyses. Very high doses of soy or soy protein were used in previous intervention studies to evaluate potential beneficial effects (Duncan et al, 1999; Lu et al, 2000; Pino et al, 2000). On the other hand, the majority of analytical epidemiologic studies reported thus far used women who consumed none or very low amount of soyfoods as the reference group (Hirayama, 1990; Lee et al, 1991; Hirose et al, 1995; Greenstein et al, 1996; Wu et al, 1996; Witte et al, 1997; Key et al, 1999). Most soy isoflavonoids are excreted in urine within 96 hours after soyfood consumption (Kelly et al, 1995), indicating that it requires women to eat soyfoods at least on a weekly basis to maintain a relatively constant isoflavonoid level in their body. It is possible that no additional beneficial effect may be seen with increasing intake amount of soyfoods among regular (weekly) soy eaters. An alternative explanation may be that the precise intake levels of soyfoods cannot be measured optimally among women who consumed high levels of soyfoods. This is less likely, however, since as noted above, we have shown that the validity of the FFQ in assessing usual soyfood intake is high.

Our findings for a strong association of soyfoods with breast cancer positive for both ER and PR and a potential modifying effect of BMI are supported by previous in vivo and in vitro studies (Adlercreutz and Mazur, 1997; Wagner et al, 1997). Soy isoflavones have a diphenolic structure similar to that of oestrogens and have been shown to have a weak oestrogenic and anti-oestrogenic activity in many in vitro and in vivo systems (Adlercreutz and Mazur, 1997). The anti-cancer effects have been suggested to be mediated through various mechanisms, including competing with endogenous oestrogens in binding to the oestrogen receptors and nuclear oestrogen-binding sites, decreasing blood levels of oestrogens and free oestrogens by increasing SHBG, and inhibiting important steroid biosynthetic enzymes (Adlercreutz and Mazur, 1997). After the menopause, adipose tissue is the major site for oestrogen synthesis and women with a high BMI have an elevated level of endogenous oestrogens (Siiteri, 1987). A number of studies have found that high BMI is associated with an elevated risk of ER+ breast cancer (Potter et al, 1995). Body weight and central obesity have also been associated with an elevated level of insulin and insulin-like growth factors (IGFs) (Pasquali et al, 1987; Seidell, 2000). Insulin resistance and an elevated level of IGF-I have been shown to be associated with an increased risk of breast cancer (Bruning et al, 1992). Oestradiol may also work synergistically with IGFs in the aetiology of breast cancer (Thorsen et al, 1992). In addition to anti-oestrogenic effects, several recent animal studies have shown that soy protein improves glucose tolerance and insulin sensitivity (Wagner et al, 1997).

In summary, our study showed that regular soyfood intake, especially at very high level, may be associated with a reduced risk of breast cancer, particularly for those positive for ER and PR. The potential protective effect may be more pronounced in women with a high BMI. These findings were biologically plausible and consistent with cancer-inhibiting effects of soy and its constituents. Our finding, if confirmed, may have implications for the primary prevention of breast cancer.

© 2001 Cancer Research Campaign 

### ACKNOWLEDGEMENTS

This work was supported by USPHS Grant R01 CA64277.

### REFERENCES

Adlercreutz H and Mazur W (1997) Phyto-oestrogens and Western diseases. *Ann Med* **29**: 95–120

Breslow NE and Day NE (1980) *Statistical methods in cancer research. The analysis of case-control studies*. Lyon (France): International Agency for Research on Cancer

Bruning PF, Bonfrer JM, van Noord PA, Hart AA, Jong-Bakker M and Nooijen WJ (1992) Insulin resistance and breast-cancer risk. *Int J Cancer* **52**: 511–516

Chen Z, Zheng W, Custer LJ, Dai Q, Shu XO, Jin F and Franke AA (1999) Usual dietary consumption of soy foods and its correlation with the excretion rate of isoflavonoids in overnight urine samples among Chinese women in Shanghai. *Nutr Cancer* **33**: 82–87

Chinese Academy of Medical Sciences (1991) *Food Composition Tables*. Beijing: People's Health Publishing House

Constantinou AL, Rossi H, Nho CW, Jefferey EH, Xu Xy, Breemen RBV and Pezzuto JM (2000) Soy protein isolate depleted fo isoflavones prevents DMBA-induced mammary tumors in female rats. *AACR meeting*

Duncan AM, Underhill KE, Xu X, Lavalleur J, Phipps WR and Kurzer MS (1999) Modest hormonal effects of soy isoflavones in postmenopausal women [published erratum appears in J Clin Endocrinol Metab 2000 Jan; 85(1): 448]. *J Clin Endocrinol Metab* **84**: 3479–3484

Greenstein J, Kushi L, Zheng W, Fee R, Campbell D, Campbell D, Sellers T and Folsom A (1996) Risk of breast cancer associated with intake of sepcific foods and food groups. *Am J Epidemiol* **143**: S36

Hankinson SE, Willett WC, Colditz GA, Hunter DJ, Michaud DS, Deroo B, Rosner B, Speizer FE and Pollak M (1998) Circulating concentrations of insulin-like growth factor-I and risk of breast cancer [see comments]. *Lancet* **351**: 1393–1396

Henderson BE, Pike MC, Bernstein L and Whelen SL (1996) Breast Cancer. In *Cancer Epidemiology and Prevention*, Schottenfeld D, Parkin DM (eds) pp 1022–1039. Oxford University Press: New York

Hirayama T (1990) Life-style and mortality: a large-scale census-based cohort study in Japan. Wahrendorf J (ed) Basel: Karger

Hirohata T, Shigematsu T, Nomura AM, Nomura Y, Horie A and Hirohata I (1985) Occurrence of breast cancer in relation to diet and reproductive history: a case-control study in Fukuoka, Japan. *Natl Cancer Inst Monogr* **69**: 187–190

Hirose K, Tajima K, Hamajima N, Inoue M, Takezaki T, Kuroishi T, Yoshida M and Tokudome S (1995) A large-scale, hospital-based case-control study of risk factors of breast cancer according to menopausal status. *Jpn J Cancer Res* **86**: 146–154

Kelly GE, Joannou GE, Reeder AY, Nelson C and Waring MA (1995) The variable metabolic response to dietary isoflavones in humans. *Proc Soc Exp Biol Med* **208**: 40–43

Key TJ, Sharp GB, Appleby PN, Beral V, Goodman MT, Soda M and Mabuchi K (1999) Soya foods and breast cancer risk: a prospective study in Hiroshima and Nagasaki, Japan. *Br J Cancer* **81**: 1248–1256

Lee HP, Gourley L, Duffy SW, Esteve J, Lee J and Day NE (1991) Dietary effects on breast-cancer risk in Singapore [see comments]. *Lancet* **337**: 1197–1200

Lee HP, Gourley L, Duffy SW, Esteve J, Lee J and Day NE (1992) Risk factors for breast cancer by age and menopausal status: a case-control study in Singapore. *Cancer Causes Control* **3**: 313–322

Lu LJ, Anderson KE, Grady JJ, Kohen F and Nagamani M (2000) Decreased ovarian hormones during a soya diet: implications for breast cancer prevention. *Cancer Res* **60**: 4112–4121

Messina M, Barnes S and Setchell KD (1997) Phyto-oestrogens and breast cancer [comment]. *Lancet* **350**: 971–972

Parkin DM, Whelen SL, Ferlay J and Raymond L (1997) *Cancer Incidence in Five Continents*. Lyon: International Agency for Research on Cancer

Pasquali R, Antenucci D, Melchionda N, Fabbri R, Venturoli S, Patrono D and Capelli M (1987) Sex hormones in obese premenopausal women and their relationships to body fat mass and distribution, B cell function and diet composition. *J Endocrinol Invest* **10**: 345–350

Pino AM, Valladares LE, Palma MA, Mancilla AM, Yanez M and Albala C (2000) Dietary isoflavones affect sex hormone-binding globulin levels in postmenopausal women. *J Clin Endocrinol Metab* **85**: 2797–2800

Potischman N, Swanson CA, Coates RJ, Weiss HA, Brogan DR, Stanford JL, Schoenberg JB, Gammon MD and Brinton LA (1997) Dietary relationships with early onset (under age 45) breast cancer in a case-control study in the United States: influence of chemotherapy treatment [see comments]. *Cancer Causes Control* **8**: 713–721

Potischman N, Swanson CA, Coates RJ, Gammon MD, Brogan DR, Curtin J and Brinton LA (1999) Intake of food groups and associated micronutrients in relation to risk of early-stage breast cancer. *Int J Cancer* **82**: 315–321

Potter JD, Cerhan JR, Sellers TA, McGovern PG, Drinkard C, Kushi LR and Folsom AR (1995) Progesterone and estrogen receptors and mammary neoplasia in the Iowa Women's Health Study: how many kinds of breast cancer are there? *Cancer Epidemiol Biomarkers Prev* **4**: 319–326

Satia JA, Patterson RE, Herrero R, Jin F, Dai Q, King IB, Chen C, Kristal AR, Prentice RL and Rossing MA (1999) Study of diet, biomarkers and cancer risk in the United States, China and Costa Rica. *Int J Cancer* **82**: 28–32

Seidell JC (2000) Obesity, insulin resistance and diabetes–a worldwide epidemic. *Br J Nutr* 83 Suppl 1: S5–S8

Siiteri PK (1987) Adipose tissue as a source of hormones. *Am J Clin Nutr* **45**: 277–282

Thorsen T, Lahooti H, Rasmussen M and Aakvaag A (1992) Oestradiol treatment increases the sensitivity of MCF-7 cells for the growth stimulatory effect of IGF-I. *J Steroid Biochem Mol Biol* **41**: 537–540

Wagner JD, Cefalu WT, Anthony MS, Litwak KN, Zhang L and Clarkson TB (1997) Dietary soy protein and estrogen replacement therapy improve cardiovascular risk factors and decrease aortic cholesteryl ester content in ovariectomized cynomolgus monkeys. *Metabolism* **46**: 698–705

Willett WC (1998) *Nutritional Epidemiology: Monographs in Epidemiology and Biostatistics*. Oxford: New York

Witte JS, Ursin G, Siemiatycki J, Thompson WD, Paganini-Hill A and Haile RW (1997) Diet and premenopausal bilateral breast cancer: a case-control study. *Breast Cancer Res Treat* **42**: 243–251

Wu AH, Ziegler RG, Horn-Ross PL, Nomura AM, West DW, Kolonel LN, Rosenthal JF, Hoover RN and Pike MC (1996) Tofu and risk of breast cancer in Asian-Americans. *Cancer Epidemiol Biomarkers Prev* **5**: 901–906

Wu AH, Ziegler RG, Nomura AM, West DW, Kolonel LN, Horn-Ross PL, Hoover RN and Pike MC (1998) Soy intake and risk of breast cancer in Asians and Asian Americans. *Am J Clin Nutr* **68**: 1437S–1443S

Yang G, Zheng W, Lu G, Dai Q, Best R, Gao YT, SHu XO, Shrubsole M and Jin F (2001) *Am Ass Cancer Res*

Yuan JM, Wang QS, Ross RK, Henderson BE and Yu MC (1995) Diet and breast cancer in Shanghai and Tianjin, China. *Br J Cancer* **71**: 1353–1358

Zheng W, Dai Q, Custer LJ, Shu XO, Wen WQ, Jin F and Franke AA (1999) Urinary excretion of isoflavonoids and the risk of breast cancer. *Cancer Epidemiol Biomarkers Prev* **8**: 35–40

© 2001 Cancer Research Campaign
